# Genomic Surveillance of SARS-CoV-2 Variants Circulating in Rajasthan in 2025

**DOI:** 10.7759/cureus.109663

**Published:** 2026-05-26

**Authors:** Abhaya Sharma, Swati Gautam, Pratibha Sharma, Farah Deeba, Himanshu Sharma, Neha Bhomia, Nita Pal, Ruchi Singh, Ravi P Sharma, Sanjay Deshpande, Varsha Potdar, Veena Vipat, Shweta Choudhary, Bharti Malhotra

**Affiliations:** 1 Microbiology, Sawai Man Singh Medical College, Jaipur, IND; 2 Public Health, Directorate of Medical and Health Services, Jaipur, IND; 3 Infectious Disease, Indian Biological Data Centre, Regional Centre for Biotechnology, Faridabad, IND; 4 Infectious Disease, Indian Council of Medical Research (ICMR), National Institute of Virology (NIV), Pune, IND; 5 Pediatrics, BJ Government Medical College (BJGMC), Pune, IND; 6 Infectious Disease, Influenza Group Division, National Institute of Virology (NIV), Pune, IND

**Keywords:** insacog, next generation sequencing (ngs), real time pcr, sars-cov-2 and covid-19, severe acute respiratory illness (sari)

## Abstract

Introduction: The COVID-19 pandemic, caused by the SARS-CoV-2 virus, has undergone significant evolutionary changes since its onset, resulting in the emergence of multiple variants with distinct genetic profiles and public health implications. Numerous genetic modifications have occurred in the virus since it first appeared, leading to the development of several variants with varying degrees of virulence, transmissibility, and immunological escape potential.

Materials and methods: A total of 602 throat/nasopharyngeal swabs of SARS-CoV-2-positive patients were received at Sawai Man Singh Medical College (SMSMC), Jaipur, India, from April 30, 2025, to September 5, 2025, for next-generation sequencing, of which 373 samples having a Ct value ≤ 25 were further processed for sequencing.

Results: Among the 373 lineages identified, XFG was the predominant lineage and was detected in 182 (48.8%) cases, including​​​​​​​ XFG (85), XFG.3 (95), and XFG.3.1 (2). Other lineages identified were PY.1 (65, 17.43%), LF.7 and its sublineages (61, 16.35%), JN.1 and its sublineages (23, 6.17%), PY.2 (23, 6.17%), BA.2 and its sublineages (8, 2.14%), PL.1.1 (3, 0.8%), LP.8.1 (2, 0.53%), NB.1.8.1 (2, 0.53%), XFC.1 (1, 0.27%), XFJ (1, 0.27%), PQ.7 (1, 0.27%) and IN.1.16.1 (1, 0.27%). Among the total positive cases identified, 55.76% were males and 44.24% were females. Regarding age distribution, 10.19% were aged <18 years, 68.90% were aged 19-59 years, and 20.91% were aged ≥60 years. Overall, 21.18% of cases were asymptomatic, whereas 78.82% were symptomatic. Reinfection was observed in 28.95% (108) of cases. ​​​​​​​​​​​​​​​​​​Most positive cases were reported from the Jaipur district. The majority of positive cases (88.47%, 330) had already been vaccinated. Comorbidities or other underlying conditions were present in 25.74% (96) of patients. Hospitalization was required in 25.47% (95) of cases, and death due to COVID was reported in three cases.

Conclusion: The highest upsurge in COVID cases during the study period was due to the XFG variant, which is a recombinant hybrid of the LF.7 and LP.8.1.2 Omicron subvariants. Continuous genomic surveillance helps in detecting real-time viral recombination and mapping spike protein mutations that lead to the emergence of such variants. Genomic surveillance revealed a wide range of SARS-CoV-2 variants in Rajasthan, with implications for public health response and vaccination strategies. The study underscores the critical importance of sustained genomic surveillance programs in Rajasthan to track viral evolution in near real-time, mitigate transmission risks, and strengthen pandemic preparedness at both regional and national levels.

## Introduction

During the SARS-CoV-2 pandemic, numerous variants have emerged [1], and due to the ability of the virus to accumulate mutations, they will continue to appear as long as the virus exists. Indeed, SARS-CoV-2 has gathered numerous mutations over time, leading to the development of multiple lineages and sublineages, each exhibiting different levels of transmissibility [2]. Through the study of viral genomes, scientists can identify mutations that might influence transmissibility, immune escape, and the effectiveness of vaccines. This knowledge is crucial for modifying public health actions, refreshing vaccines, and executing focused interventions to manage the spread of the virus​​​.

SARS-CoV-2 continues to undergo genetic changes, and notable shifts in global variant patterns occurred between January and May 2025. Early in the year, WHO data indicated that XEC was the dominant circulating variant worldwide, with KP.3.1.1 following closely behind. By February, however, XEC detections began to decrease, while LP.8.1 showed a steady increase and eventually became the most frequently identified variant by mid-March. From mid-April onward, LP.8.1 displayed a slight downturn in circulation as NB.1.8.1 started gaining prominence across global sequences [3]. XFG variant, which emerged in early 2025, has been associated with increased transmissibility due to specific mutations in the spike protein, underscoring the importance of vigilant genomic surveillance to detect and respond to emerging strains promptly.

The present study was conducted to monitor emerging SARS-CoV-2 variants through genomic surveillance in the state of Rajasthan. Genomic surveillance is essential for monitoring the evolution of SARS-CoV-2, recognizing new variants, and guiding public health approaches. A robust genomic surveillance was implemented in Rajasthan by the state health department, ensuring the timely detection of variants and the monitoring of mutation patterns for strengthened evidence-based public health response and preparedness across the state.

## Materials and methods

Study design

This is a cross-sectional, laboratory-based genomic surveillance study.

Sample size calculation

The sample size was calculated using the formula n = Z²pq/d², assuming a prevalence of 50%, a 95% confidence level, and 5% precision, yielding a minimum required sample size of 384. The study analyzed 373 samples, which was comparable to the calculated sample size.

Sample collection

A total of 602 throat and nasopharyngeal swab samples from COVID-19-positive patients, collected in viral transport medium (VTM), were received from all districts of Rajasthan at the Department of Microbiology, Sawai Man Singh (SMS) Medical College, Jaipur, India, between April 30, 2025, and September 5, 2025, for whole-genome sequencing, in accordance with state government directives to sequence all positive cases.

Nucleic acid extraction and real-time polymerase chain reaction (PCR)

Nucleic acid extraction was performed using an automated extraction system, NucliSens easyMAG (BioMérieux, France), using 400 μl VTM. A repeat PCR was performed on all 602 samples, of which 373 with Ct values ≤ 25 were further processed for next-generation sequencing (NGS).

Genome sequencing and analysis

Manual library preparation was performed using the Ion AmpliSeq Library Kit Plus (Invitrogen, USA) along with the Ion AmpliSeq SARS-CoV-2 Research Assay Panel (Life Technologies, CA). The Ion Chef Automated System (Life Technologies, CA) was used for template preparation, enrichment, and chip loading. Sequencing was performed on the Ion Torrent S5 Plus system (Thermo Fisher Scientific, USA), which detects the release of hydrogen ions when a new nucleotide is incorporated into the expanding DNA template, enabling quick, cost-effective, and scalable sequencing.The Torrent Suite software v5.12.0 (Thermo Fisher Scientific, USA) was used to analyze the data. For lineage determination, the FASTQ files were submitted to IBDC (Indian Biological Data Centre), and the FASTA files were downloaded from the IBDC portal. FASTA sequences containing <1% unknown nucleotides (N) were considered valid. Phylogenetic trees were constructed using Nextclade with Wuhan-Hu-1/2019 (GenBank: MN908947) as a reference for alignment and were visualized using the Nextstrain Auspice (Nextstrain, Seattle, WA) platform (last accessed on December 15, 2025). The study received ethical approval from the Ethics Committee of SMS Medical College (Ref. No.: 299/MC/EC/2022 dated April 28, 2022).

Statistical analysis

The demographic details of the patients were obtained through a filled proforma. Clinical data were collected either telephonically or through IDSP. The demographic and clinical details were entered in MS Excel (Microsoft Corporation, Redmond, WA). Categorical variables were reported as numbers and percentages. GraphPad Prism (online version; GraphPad, San Diego, CA) was used for statistical analysis. Chi-square test was applied to calculate p-values for categorical variables at a 95% confidence interval. A p-value ≤ 0.05 was considered statistically significant.

## Results

**Figure 1:**
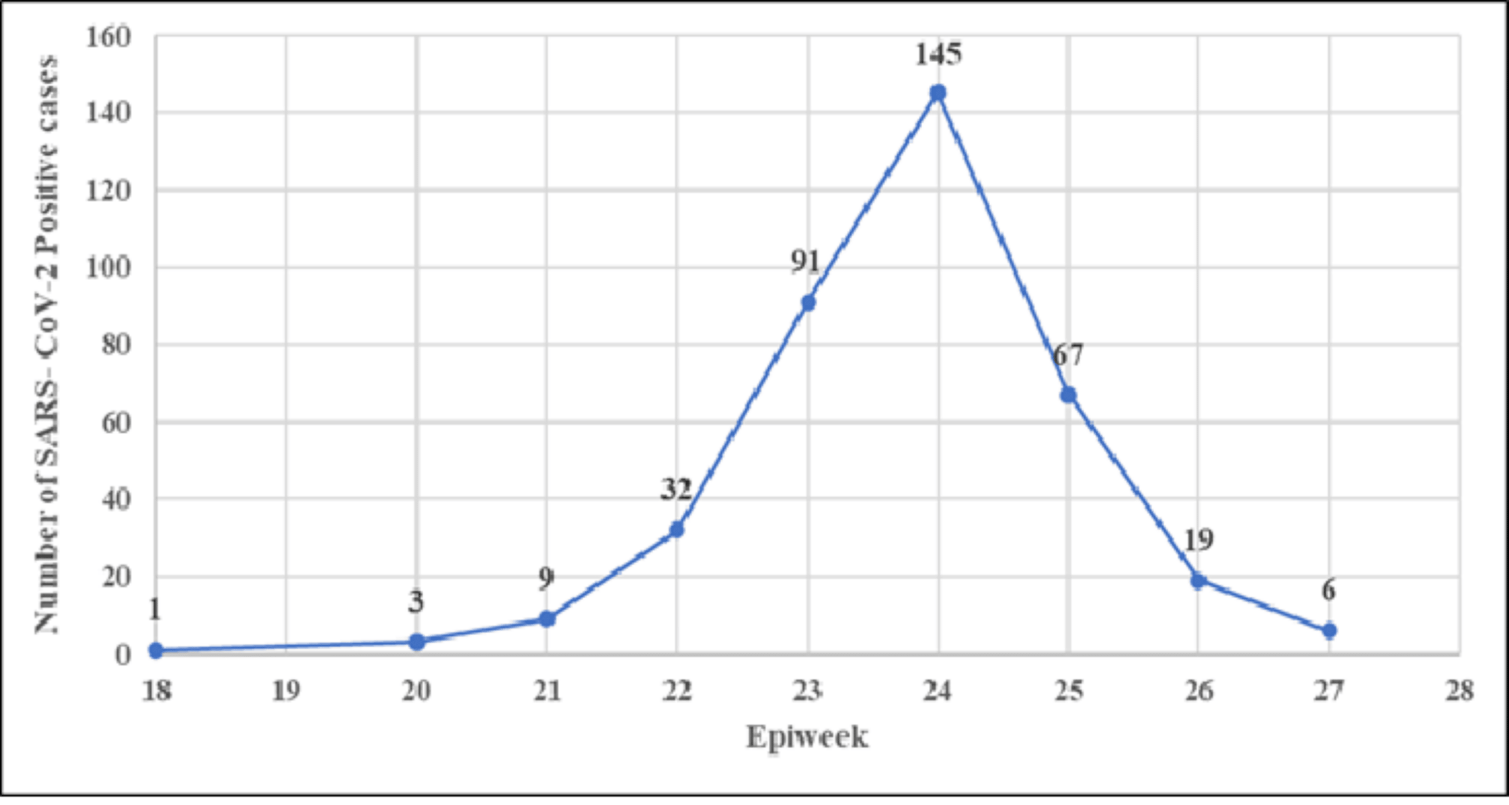
Epiweek-wise emergence of SARS-CoV-2 positive cases

The number of SARS-CoV-2-positive cases increased significantly from epidemiological week (epiweek) 18 to epiweek 27, as shown in Figure 1.

**Figure 2:**
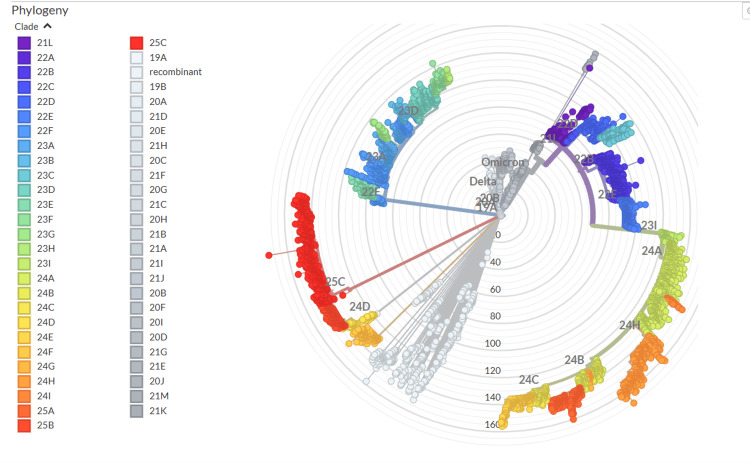
Phylogenetic representation of the SARS-CoV-2 lineages

A total of 373 samples met quality scores and achieved over 95% genome coverage. Of all the lineages identified, XFG was the predominant lineage, which was detected in 182 (48.8%) cases (XFG (85, 22.78%), XFG.3 (95, 25.46%), XFG.3.1 (2, 0.53%)). Other lineages identified were PY.1 (65, 17.43%), LF.7 and its sublineages (61, 16.35%), JN.1 and its sublineages (23, 6.17%), PY.2 (23, 6.17%), BA.2 and its sublineages (8, 2.14%), PL.1.1 (3, 0.8%), LP.8.1 (2, 0.53%), NB.1.8.1 (2, 0.53%), XFC.1 (1, 0.27%), XFJ (1, 0.27%), PQ.7 (1, 0.27%), and IN.1.16.1 (1, 0.27%). The phylogenetic tree was constructed, downloaded, and visualized using Nextstrain Auspice (accessed on December 15, 2025) web software (Figure 2).

Of the total positive cases, 208 (55.76%) were males, and 165 (44.24%) were females. The majority of cases (257, 68.90%) belonged to the 19-59-year age group, followed by the ≥60-year age group (78, 20.91%). Symptoms were absent in 79 (21.18%) cases, while 294 (78.82%) cases were symptomatic. The most common symptoms were cold (172, 58.5%), fever (138, 46.93%), cough (120, 40.81%), sore throat (70, 23.80%), rhinorrhea (70, 23.8%), breathlessness (66, 22.44%), nausea (13, 4.42%), body pain (11, 3.74%), chest pain (9, 3.40%), headache (7, 2.38%), and pneumonia (5, 1.70%). The majority of SARS-CoV-2-positive cases (330, 88.47%) had a history of vaccination, with most having received two vaccine doses. Among the vaccinated individuals, 260 (78.79%) were symptomatic, whereas 70 (21.21%) were asymptomatic. Of the 43 unvaccinated cases, five (11.63%) were unvaccinated adults, while 38 (88.37%) were not eligible for vaccination (age ≤ 18 years).

No significant differences were observed between vaccinated and unvaccinated cases with respect to various clinical symptoms, including fever, cough, rhinorrhea, sore throat, breathlessness, nausea, body pain, chest pain, and headache (p > 0.05). However, cold and pneumonia were significantly more common among unvaccinated cases (p < 0.05), as shown in Table 1.

**Table 1:** Clinical characteristics and outcomes of COVID-19 infections among vaccinated and unvaccinated cases

Parameters	Total (Percentage)	Vaccinated (N = 330)	Unvaccinated (N = 43)	P-value
Clinical characteristics of COVID-19 patients				
Asymptomatic	79 (21.18)	70 (21.21)	9	0.966
Symptomatic	294 (78.82)	260 (78.79)	34	
Common symptoms				
Cold	172 (58.5)	143	29	0.002
Fever	138 (46.93)	119	19	0.299
Cough	120 (40.81)	109	11	0.325
Sore throat	70 (23.8)	60	10	0.422
Rhinorrhea	66 (22.44)	55	11	0.149
Breathlessness	14 (4.76)	10	4	0.041
Nausea	13 (4.42)	11	2	0.657
Body pain	11 (3.74)	8	3	0.097
Chest pain	9 (3.4)	7	2	0.309
Headache	7 (2.38)	5	2	0.154
Pneumonia	5 (1.7)	2	3	<0.001
Clinical outcome				
Hospitalization	95 (25.47)	74	21	<0.001
Home isolation	278 (74.53)	256	22	
Comorbidities				
With one or more	96 (25.74)	79	17	
Diabetes	36 (37.5)	32	4	0.934
Hypertension	32 (33.33)	30	2	0.328
Heart disease	10 (10.41)	7	3	0.063
Thyroid	5 (5.20)	4	1	0.55
Asthma	5 (5.20)	3	2	0.044
Chronic lung disease	4 (4.16)	3	1	0.396
Cancer	4 (4.16)	2	2	0.015
Chronic renal disease	2 (2.08)	1	1	0.087
Liver disease	2 (2.08)	2	0	0.608
Pregnancy	2 (2.08)	2	0	0.608
Tuberculosis	2 (2.08)	1	1	0.087
Lab-confirmed reinfection				
Yes	108 (28.95)	90	18	0.047
No	265 (71.05)	240	25	

About 74.26% (277) of cases had no comorbidities, while 25.74% (96) had one or more comorbidities/other conditions. The most common comorbid conditions were diabetes (36, 37.50%), hypertension (32, 33.33%), heart disease (10, 10.41%), thyroid disorders (5, 5.20%), chronic lung disease (4, 4.16%), chronic renal disease (2, 2.08%), liver disease (2, 2.08%), pregnancy (2, 2.08%), and tuberculosis (2, 2.08%). These conditions did not differ significantly between vaccinated and unvaccinated cases (p > 0.05), except for asthma (5, 5.20%) and cancer (4, 4.16%), which were significantly higher in unvaccinated cases (p < 0.05) (Table 1). Of the 95 hospitalized cases, 36 (37.89%) had other medical conditions and were admitted for surgery but were found to be positive for COVID during prior testing. Cases without a history of vaccination had a considerably greater hospitalization rate compared with vaccinated cases (p < 0.05). Of the total cases, 28.95% (108) were reinfections, whereas 71.05% (265) were first-time infections. Reinfection was significantly higher in unvaccinated cases (p < 0.05) (Table 1). Death due to COVID-19 was reported in three cases. Jaipur district reported the highest number of positive cases (221, 59.24%), followed by Udaipur (42, 11.26%).

## Discussion

In May 2025, there was a sudden increase in COVID-positive cases in India. Following this, the Indian government advised states to regularly report and monitor cases of severe acute respiratory infection (SARI) and influenza-like illness (ILI) district-wise and to ensure sufficient testing for sending positive samples to the Indian SARS-CoV-2 Consortium on Genomics (INSACOG) laboratories for genome sequencing to detect new variants on time. Following this advisory, COVID-positive samples from across Rajasthan were received at the Department of Microbiology, SMS Medical College, Jaipur, India.

Cases started to increase rapidly in 2025 from week 20, peaked around week 24, and subsequently declined. Among all the identified lineages, XFG was the predominant lineage, which was detected in 182 (48.8%) cases (XFG (85, 22.78%), XFG.3 (95, 25.46%), and XFG.3.1 (2, 0.53%)). The predominance of the XFG lineage in Rajasthan may be attributed to a combination of viral genetic advantages, regional population immunity dynamics, and local transmission factors. Similarly, XFG.3 (45.27%) and XFG.4 (41.78%) were the predominant lineages found in the study by Karyakarte et al. [4]. The recombinant XFG variant, derived from LF.7 and LP.8.1.2, carries four key spike mutations (H445R, N487D, Q493E, and T572I) and has spread rapidly worldwide since it was first identified in Canada [5]. XFG was among the seven variants under monitoring (VUMs) tracked by the WHO and was classified as a VUM on June 25, 2025 [6,7]. By June 22, 2025, a total of 1648 XFG sequences from 38 countries had been submitted to GISAID [8], accounting for 22.7% of worldwide sequences reported during epidemiological week (EW) 22 of 2025 (May 26 to June 1, 2025).

However, the number of reported cases does not accurately reflect infection rates due to decreased testing and reporting worldwide. Hospitalization and death statistics are viewed as more dependable measures for assessing the burden of COVID-19 than case reporting alone. In our study, hospitalization was reported in 25.47% of cases; however​​​​, it should be noted that this data not only includes COVID-19 cases but also the hospitalized incidental cases of SARS-CoV-2 infection. There was a significant difference in hospitalization rates between vaccinated and unvaccinated cases, with the majority being vaccinated individuals. Earlier studies have reported that vaccination reduces disease severity, although it may not completely prevent infection. In our study, infections caused by these SARS-CoV-2 variants were predominantly mild in nature. Most affected individuals recovered fully without requiring hospitalization, with symptoms resolving under home isolation and treatment. These findings suggest that the variants under investigation were not associated with increased disease severity compared with earlier circulating variants [9].

Additionally, 78.82% of the infected individuals in the current study were symptomatic​​​​​​, whereas 21.18% were asymptomatic​​​​​​. Prior tests revealed that asymptomatic patients who visited the hospital's outpatient department (OPD) for various conditions were positive for SARS-CoV-2. Most symptomatic cases had minor symptoms. In the present study, one or more comorbid conditions were present in 25.74% cases. In our study, death due to COVID was ascertained in three cases.

Limitations of the study

The selection of samples with low Ct values (Ct ≤ 25) might have created a bias in the present study. Lack of longitudinal follow-up of the patients was another limitation. Moreover, the samples included in the present study were limited to Rajasthan, which may limit the generalizability of the findings.

## Conclusions

The emergence and replacement of viral variants are natural parts of a virus’s evolutionary process. Nevertheless, this should not result in reduced vigilance or decreased efforts to track new variants. In our study, SARS-CoV-2 variants were predominantly mild in nature, and the majority of affected individuals recovered fully without requiring hospitalization. Current findings offer only a momentary view of the situation, and as the virus continues to evolve, ongoing and comprehensive genomic surveillance remains crucial for detecting key genetic changes and supporting prompt and effective public health actions. Identifying specific mutations allows the design of boosters that precisely target the most current immune-evasive strains. Moreover, linking genomic variants to milder clinical outcomes helps to accurately predict hospital bed and oxygen demands during a lineage replacement event.
